# Modern Trends in the In Vitro Production and Use of Callus, Suspension Cells and Root Cultures of Medicinal Plants

**DOI:** 10.3390/molecules25245805

**Published:** 2020-12-09

**Authors:** Olga Babich, Stanislav Sukhikh, Artem Pungin, Svetlana Ivanova, Lyudmila Asyakina, Alexander Prosekov

**Affiliations:** 1 Institute of Living Systems, Immanuel Kant Baltic Federal University, A. Nevskogo Street 14, 236016 Kaliningrad, Russia; olich.43@mail.ru (O.B.); stas-asp@mail.ru (S.S.); apungin@kantiana.ru (A.P.); 2 Department of Bionanotechnology, Kemerovo State University, Krasnaya Street 6, 650043 Kemerovo, Russia; alk_kem@mail.ru; 3 Natural Nutraceutical Biotesting Laboratory, Kemerovo State University, Krasnaya Street 6, 650043 Kemerovo, Russia; 4 Department of General Mathematics and Informatics, Kemerovo State University, Krasnaya Street 6, 650043 Kemerovo, Russia; 5 Laboratory of Biocatalysis, Kemerovo State University, Krasnaya Street 6, 650043 Kemerovo, Russia; a.prosekov@inbox.ru

**Keywords:** medicinal plants, biologically active substances, callus and suspension cultures, root cultures, *Agrobacterium*

## Abstract

This paper studies modern methods of producing and using callus, suspension cells and root cultures of medicinal plants in vitro. A new solution for natural product production is the use of an alternative source of renewable, environmentally friendly raw materials: callus, suspension and root cultures of higher plants in vitro. The possibility of using hairy root cultures as producers of various biologically active substances is studied. It is proven that the application of the genetic engineering achievements that combine in vitro tissue culture and molecular biology methods was groundbreaking in terms of the intensification of the extraction process of compounds significant for the medical industry. It is established that of all the callus processing methods, suspension and root cultures in vitro, the *Agrobacterium* method is the most widely used in practice. The use of agrobacteria has advantages over the biolistic method since it increases the proportion of stable transformation events, can deliver large DNA segments and does not require special ballistic devices. As a result of the research, the most effective strains of agrobacteria are identified.

## 1. Introduction

Since ancient times, many widespread and rare plant species have been used by the population and in medicine to treat various diseases. Preference is always given to natural products as the most balanced in biochemical composition and less hazardous for use. Unfortunately, the share of new medicinal substances with high physiological activity and new product forms obtained from plant materials did not exceed 24% by the beginning of the twenty-first century [1,2].

The use of plants is constrained by the lack of technologies for the effective production of biologically active substances from plant raw materials, the poor development of cultivated botanical medicinal plant species in farms of different forms of ownership, even in botanical gardens, and by the lack of trained personnel in this direction. In addition, over the past 50 years, the number of species structures of the plant population in the natural environment, including medicinal ones, has significantly decreased [3]. This was negatively influenced by the high anthropogenic and technogenic load on biogeocenoses and by the increasing number of “hot spots”, which, according to scientists, will lead to the complete loss of 8% of various plant species in the next 25 years. The process of global desertification has affected the territories of 110 countries of the world (World Resources Institute). Many, especially endemic, species have disappeared; some remain small in numbers and are under threat of extinction [4]. Their inclusion in the Russian and regional Red Lists not only increases the medical demand for substances but also the demand for effective technologies by the pharmaceutical industry, the appearance of which is impossible without systematic research on reproduction, a return to favorable habitats and the expansion of the range of rare medicinal plants [5,6]. Therefore, medicinal plants have to be excluded from the objects of research, although many of them are the only sources of production of medicinal substances that are practically unique in their value for the treatment of oncological, neurological, Alzheimer’s and many other diseases. These include, for example, the flavones of *Scutellaria baicalensis*; in particular, vogonosin, which is characterized by high apoptotic properties and allows the targeted destruction of cancer cells without affecting healthy ones. This species can be extracted from the natural environment in very small quantities, considering its low bioavailability, and medicinal substances can be produced only through the use of a biomaterial by methods of cellular bioengineering [7].

Recently, medicinal plants, as producers of secondary synthesis substances that can affect microorganisms, animals and humans in different ways, have become of particular interest. They are aimed not only at maintaining general health but also at targeted use in the treatment of the most serious diseases, such as diseases of the cardiovascular system, cancers, diabetes, etc.

In general, the state of the population’s health reflects the need for the development of domestic science at a more modern level, including the use of all possible reserves in the development of natural components from medicinal plant species to obtain biologically active substances (compounds isolated from plants) and their targeted use in the treatment of serious diseases. The Government of the Russian Federation allocated 1 trillion rubles from the country’s budget for the construction and equipment of cancer centers until 2024, as well as 70 billion rubles to fight cancer in 2019, 120 billion in 2020, and 40 billion in 2021. A fairly large amount of funding has been allocated for innovative research and the development of promising technologies for obtaining biologically active substances from medicinal plant species. The elite group of the most important anti-cancer plants includes ginseng (*Ginseng quinquefolium*), aralia (*Aralia elata*) and *Eleutherococcus* [5]. The especially high demand for these plants in the modern market for plant materials is due to their antioxidant properties and the membrane-stabilizing effect of polyphenolic compounds [8]. Their properties and the demand for them in anticancer therapy are well known since the 1980s [9].

The most widespread use of natural products is noted in the United States, where almost 55% of the population uses herbs for treatment. In the Russian Federation, their share in pharmacies is up to 30% of all drugs. The volume of the market for medicinal plants in Russia is 11–12 million US dollars or 0.5–1.5% of the total volume of the pharmaceutical market.

Of the 250,000–500,000 species of higher vascular plants on the planet, about 80,000 are of medicinal value, and only 0.5% of them have been screened for medicinal properties [7]. One of the advantages of obtaining biologically active substances from plants is their wide range of activity and the ecological safety of their production [7]. At the same time, the use of medicinal plants in the future might be significantly limited due to the problem of depletion of biological resources.

It is expected that the number of endangered plant species will increase from 18 thousand at present to 60 thousand by the middle of the twenty-first century. Medicinal plants will make up a large part of these, as they are a particularly vulnerable category due to intensive, irrational, and insufficiently controlled procurement of raw materials [1]. Many plant species that are valuable for medicine are included in the Red Lists of the Russian Federation and its regions, and it is banned to remove them from the natural environment. Therefore, it is important not only to avoid disturbing the existing biocenoses but also to use methods for preserving rare and endangered flora representatives.

Callus, plant cell and tissue suspension cultures in vitro can become alternative sources for obtaining biologically valuable substances of plant origin. Biotechnological approaches allow a product to be obtained all year round regardless of external climatic and soil conditions while preserving the natural habitats of valuable medicinal plants [10].

Thus, the use of cell, tissue and organ cultures instead of intact plants will radically solve the problem of the shortage of plant raw materials of rare species that do not grow in Russia, solving the problem of import substitution independence from foreign supplies, patents and technologies. The availability of high-quality renewable plant materials will enable the creation of efficient and affordable mass−market nutraceuticals.

A new solution is the use of an alternative source of renewable, environmentally friendly raw materials: the cultures of cells and the organs (hairy roots) of higher plants [11,12].

The cell culture of higher plants is a unique, experimentally created biological system—a population of plant somatic cells. The basic research showed that in vitro cells differ significantly from the cells of an intact plant in a number of characteristics. To a large extent, this concerns the intensity of cell growth, as well as the characteristics of the synthesis and accumulation of biologically active substances in them. The cells in culture divide intensively; under optimal growing conditions, the biomass productivity of suspension cultures can be more than a gram of dry biomass per liter of medium per day. The qualitative and quantitative composition of secondary metabolites in in vitro cells can differ significantly from that in intact plants. The content of these substances in cell cultures might be lower than in intact plants, but there are also examples in which the content of biologically active substances in in vitro cells exceeded the content in the intact plants (steroidal glycosides in the cell culture of *Dioscorea deltoidea* [13], shikonin in the cell culture of *Lithospermum erythrorhizon* [14], berberine in the cell culture of *Berberis*, etc. [15]). The production of such cell cultures requires the creation of the super-producer strain, which means that both basic and applied research are required to use higher plant cell cultures as a source of biologically active substances of plant origin (secondary metabolites) [13,16].

A particular approach to solving the problem of obtaining biologically active compounds of plant origin is the creation and use of genetically modified cell cultures, transformed with a tumor-induced (Ti) plasmid from *Agrobacterium tumefaciens* or hairy roots transformed with a root-induced (Ri) plasmid from *Agrobacterium rhyzogenes*, which, as a rule, grow rather quickly in hormone-free environments, are genetically more stable and have a higher content of secondary metabolites.

The main advantages of this approach are:

- ecological purity of biomass production by the biotechnological method
- ability to produce plant biomass with specific characteristics regardless of the season, climate and weather conditions
- high rates of biomass production
- guaranteed production of pure biomass with no pesticides, herbicides, radioactive compounds or other pollutants.
- The presence of an effective, industrial super-producer strain guarantees a higher content of the target product than does an intact plant

This work aims to study current trends in the production and use of in vitro callus, suspension cells and root cultures of medicinal plants.

## 2. The History of the In Vitro Cultivation of Callus, Suspension Cells and Root Cultures

Ideas of the possibility of cultivating plant cells first appeared at the turn of the nineteenth and twentieth centuries, but it took a lot of experiments to bring them to life. The ability of plant tissue cultures to grow indefinitely was shown by the French researcher R. Gautheret and, independently, by the American researcher P. White in the 1930s. The promising topic was addressed by many scientists around the world, and significant advances were made over the next two decades. Frederick Steward, working with carrot tissue, obtained whole plants from it in 1958 [8,14]. Gautheret’s monograph “Plant Tissues Culture”, published a year later, mentions already 142 species of higher plants grown in vitro.

In Russia, technologies for the cultivation of cells of higher plants also appeared at the end of the 1950s. First of all, we should mention Raisa Georgievna Butenko (1920–2004), a corresponding member of the USSR Academy of Sciences since 1974, who in 1984, together with her colleagues, received the State Prize for “developing the fundamental foundations of cellular (genetic) plant engineering”. In the late 1950s, under her leadership, a laboratory of isolated plant tissues and organs was created at the K.A. Timiryazev Institute of Plant Physiology. Then it became the Department of Cell Biology and Biotechnology of the Institute of Fundamental Research, Russian Academy of Sciences.

The first, and perhaps the most important, task is to obtain plants or plant tissues from which useful substances can be extracted. These are called substances of secondary metabolism, in contrast to the primary metabolites, which are necessary for the plant itself and are found in the cells of all plants. These are the substances responsible for interactions with the outside world; for example, essential oil or bitterness, which scare away herbivores, or components of the scent of a flower. Biochemical pathways for the synthesis of secondary metabolites are a superstructure of the system of vital reactions. However, there are unique medicinal compounds among them.

## 3. The Use of Hairy Root Culture as a Producer of Biologically Active Substances

In the 1980s, it was proposed to use the hairy root cultures as producers of various biologically active substances, including rosmarinic acid, artemisinin, baicalin, aconitine anthraquinone and many other secondary metabolites.

Rosmarinic acid has antiviral, antibacterial, antioxidant, anti-inflammatory and anti-allergic activities. For the first time, this acid was isolated from rosemary (*Rosmarinus officinalis*) in 1958 and received the corresponding name [3]. It is present in many plants, but in vitro plant tissue cultures are usually used for isolation since the content of this acid in them is much higher than in natural plants. Japanese scientists [7] have created the roots of sweet basil with a content of rosmarinic acid of up to 14.1% of dry biomass using different strains of *A. rhizogenes*. In 1998, they used this technology on hyssop (*Hyssopus officinalis*). The yield of rosmarinic acid was 8% of the dry weight [2].

Polish researchers also demonstrated the possibility of producing rosmarinic acid in hyssop (6% by dry weight) and in *Salvia officinalis*, producing more than twice the amount of rosmarinic acid of plants not affected by genetic engineering [10].

Anthraquinones, various phenolic compounds and coumarin glycosides are extracted from the hairy roots of *Polygonum multiflorum* [4,5]. The root culture of *Polygonum tinctorium* is a raw material for the production of the widely used indigo dye [5,6].

Various aconitines are obtained from the hairy roots of *Aconitum heterophyllum*, which are present there in noticeably larger quantities than in the roots of ordinary plants of this species [2]. The root culture of common chicory (*Cichorium intybus*) is used for the production of various sesquiterpenes and coumarin [8].

An increased level of sesquiterpenes in comparison with a common plant was obtained in the hairy roots of dandelion (*Taraxacum officinale*) [1]. It is possible to apply hairy roots to belladonna (*Atropa belladonna*) for phytoremidation. During phytoremidation, the root culture in the presence of hydrogen peroxide purified water from phenol [5]. The role of peroxidase and hydrogen peroxide in the removal of phenol from a liquid medium with the help of the hairy roots of a number of species of the *Cruciferae* family is known. *Brassica juncea* roots were added to the medium, both in the live and lyophilized form [11,12].

Recently, it has been reported that the hairy roots of physalis degrade the azo dye Reactive Black 8 to non-toxic metabolites [10]. Over 300 species of plants (most of them from the cruciferous family) are known to hyperaccumulate heavy metals, mostly nickel [17]. The hairy roots of the *Thlaspi caerulensis* species of the cruciferous family can accumulate cadmium. Apart from cadmium and nickel, the horseradish root culture can accumulate uranium [11,12].

Extracts of tobacco (*Nicotiana tabacum*) roots, both wild type and containing transgenes encoding tomato peroxidase, were used for the successful destruction of 2,4-dichlorophenol [5]. The roots of carrots (*Daucus carota*), sweet potatoes (*Ipomoea batatas*) and *Solanum aviculare* accumulated large amounts of phenol and chlorophenol, purifying the culture medium from them [8]. The hairy roots of black nightshade (*Solanum ptychanthum*) degraded polychlorinated biphenyls, transforming them into a number of less dangerous compounds [9].

An even wider range of biologically active substances is held back by the absence of secondary growth of the roots. This could contribute to a greater yield of the target metabolic products since it is known that the production activity of substances of secondary origin often increases in the roots characterized by secondary growth [18]. Various methods of inducing the production of secondary metabolites in cultures of hairy roots and their secretion into the culture medium have not been sufficiently implemented in practice.

One of the problems is the need to preserve the roots for a long time without constant passaging. At the moment, many methods have been proposed for maintaining and preserving the status of the created culture. However, research in this area should continue to develop even more advanced methods of both cryostorage and the use of bioreactors.

## 4. Genetic Engineering and Biotechnology in the In Vitro Production of Callus, Suspension Cells and Root Cultures

Cultures of cells, tissues, and plant organs are increasingly popular alternative sources of valuable secondary metabolites. This is due to the limited reserves of medicinal raw materials, the impossibility of growing many species using the plantation method and the difficulties in developing methods for the chemical synthesis of a number of natural compounds [15,19].

A breakthrough in the intensification of the extraction process of compounds significant for the medical industry is the application of the achievements of genetic engineering that combine in vitro tissue culture and molecular biology methods [2,17,20].

For the first time, transgenic plants were obtained in 1982 by scientists from the Institute for Plant Breeding Research in Cologne and The Monsanto Company. As a result, the plants acquired resistance to the growth-inhibiting antibiotic kanamycin. Currently, Monsanto alone has produced more than 45,000 independent transgenic plant lines.

One of the important tasks in the genetic engineering of plants is to obtain plants resistant to viruses since, at present, there are no direct ways to combat viral infections of crops [18].

Scientists at Washington State University decided that virus resistance could be reached by introducing the genes for the coat protein of the tobacco mosaic virus into plant cells. The experiment fully confirmed this assumption: intensive synthesis of the coat protein of any virus in plant cells causes resistance to this virus. Currently, transgenic plants have been obtained that can withstand the effects of more than a dozen different viral infections. Another task is related to the protection of plants from insect pests [11].

The use of insecticides is not quite effective, firstly, because of their toxicity, and, secondly, because they are washed off the plants by rainwater. In the genetic engineering laboratories of Belgium and the USA, the introduction of genes into the plant cells that are responsible for the synthesis of insecticides of bacterial origin was successfully carried out. These genes were introduced into the cells of potatoes (*Solanum tuberosum*), tomatoes (*Solanum lycopersicum*) and cotton (*Gossypium arboreum*). The transgenic potato and tomato plants were resistant to the Colorado potato beetle (*Leptinotarsa decemlineata*); the cotton plants were resistant to various insects, including the cotton bollworm. The use of insecticides has been reduced by 40–60% [21,22,23,24].

Genetic engineers have developed transgenic plants with an extended period of fruit ripening. Such tomatoes, for example, can be harvested when ripe without fear that they will overripen during transportation [15,22,25].

Genetic engineering methods have been successfully applied to about fifty species, including apple (*Malus domestica*), plum (*Prunus subg. Prunus*), grape (*Vitis vinifera*), cabbage (*Brassica oleracea*), eggplant (*Solanum melongena*), cucumber (*Cucumis sativus*), wheat (*Triticum aestivum*), soy (*Glycine max*), rice (*Oryza sativa*), rye (*Secale cereale*) and many other agricultural plants, the cultivation of which will be facilitated in the near future thanks to genetic modifications [24,26].

The biotechnological approach has a number of advantages over the traditional use of plant raw materials, including the possibility of obtaining biomass regardless of the season and the climatic and soil conditions, the ease of extraction and purification of preparations, the enhancement of the biosynthesis of the necessary substances with the help of elicitors, process automation, etc. The ability of isolated plant cells to produce in vitro a spectrum of secondary metabolites synthesized in plants of the species in vivo is associated with totipotency, i.e., with the preservation of complete genetic information about the pathways of their biosynthesis and the possibility of its implementation [25,27].

The essence of this method lies in the transfer of heterologous genes isolated from different organisms into a new genetic environment. Since plant cells remain totipotent throughout the entire life cycle, a whole plant can be obtained from one modified cell. On the one hand, this approach makes it possible to introduce traits into plants that cannot be obtained using traditional breeding; on the other hand, genetic engineering allows the transfer of a separate gene responsible for a specific trait, which reduces the risk of losing an already established genotype [22,25,28].

Several studies have shown that the content of secondary metabolites in plants cultivated in vitro can exceed their concentration in intact plants [26,29].

Thus, in the regenerants of representatives of the genus *Crotalaria*, the content of alkaloids was higher than in the plants growing on the experimental plot. In addition, micropropagation in combination with other biotechnological techniques makes it possible to obtain genetically transformed plants and somaclonal variants and to carry out selection according to the level of biosynthesis, which is especially important when creating varieties and lines of medicinal plants [30].

Currently, the technologies for the production of valuable, secondary, biologically active substances from unorganized calli or suspension cultures are the most developed. However, in some cases, calli do not accumulate the metabolites of interest [9,28].

It is known that the pathways of biosynthesis of secondary metabolites require cooperation between cells, tissues and organs of plants at the intra- and intermolecular level; therefore, for certain stages of biosynthesis, cell differentiation is a critical factor. In such situations, it is necessary to use more differentiated tissues or organ cultures and sometimes microplants [31,32].

Thus, the choice of the best in vitro cultivation method (callus culture, organ culture or microplants) is a strategic moment in biotechnology that requires special attention. Moreover, for the maximum efficiency of in vitro systems, as a rule, it is required to develop approaches to enhance the biosynthesis of secondary metabolites [2,5,33,34,35].

*Hedysarum theinum Krasnob*. (family *Leguminosae*) is one of the cell cultures in demand for the biotechnological production of natural substances with anti-inflammatory, bactericidal, antispasmodic, immunoprotective and other properties [3,28,30]. Recently, this plant has been actively used for the production of various dietary supplements. However a large volume of production and low rapidity of renewal put these plants in danger of extinction.

The traits that can be given through genetic engineering are very diverse and are mainly limited only by the presence of the corresponding genes. They can be very conditionally divided into three groups.

The first group includes signs that are interesting to producers: resistance to various environmental factors such as herbicides, diseases, pests, drought, salinity, improved mineral nutrition and increased rooting [3,26,29,33,35,36]. The second group of traits is of interest directly to consumers: modification of the taste and aroma of fruits, an increase in the duration of their storage, a change in the color of flowers, seedlessness and an improvement in the nutritional value of plants. The third group includes plants with biofactories capable of synthesizing vaccines, enzymes, biopolymers and other useful substances [22,25,35,37,38,39,40].

The genetic engineering of plants began its rapid development with the emergence of technology for their transformation and the production of the first transgenic plant with the expression of a foreign gene in the early 1980s. This was followed by the emergence of new chimeric genes, vectors for transformation, methods of DNA delivery and plant regeneration systems.

It was shown that an antibiotic resistance marker can be used to select transformed cells from which it is subsequently possible to regenerate normal fertile plants. Already in 1986, the first field trials of transgenic plants—tobacco with the marker gene neomycin phosphotransferase II (nptII)—were carried out in the USA and France [1,36,37,41,42,43,44].

Tobacco (*Nicotiana tabacum*) has been and remains a key model plant in the development of genetically engineered technologies [45]. It was this plant for which the possibility of in vitro regeneration was first noted and a standard medium for cultivation was developed. Due to the simplicity of transformation, regeneration and the rate of subsequent development, tobacco remains the main model object for research in the field of plant genetic engineering [45].

A transgenic (or genetically modified) plant is a plant into which genome genes (“transgenes”) from other organisms have been transferred by genetic engineering. The process of gene transfer is called a genetic transformation. The main advantages of this technology in comparison with traditional breeding are the possibility of transferring only one gene, which practically does not affect the original genotype; the ability to impart traits that cannot be transferred by crossing with closely related species; and significant acceleration of the process of obtaining new genotypes [46].

There are several different methods for delivering DNA into a cell. In general, all methods of transferring DNA into plants can be divided into several large groups [47].

The first group includes natural gene transfer systems: agrobacterial and viral vectors and delivery methods.

The second group includes artificial transfer systems, which can be divided into two subgroups: direct injection of DNA by achieving permeability of protoplasts and cells by chemical (polyethylene glycol), physical (electroporation, ultrasound) or mechanical methods (silicon carbide crystals, laser); and the use of artificial vector systems, such as microparticles, microinjections or liposomes. The third group can include various methods, which, unlike the first two, do not require in vitro culture and are usually called in planta transformation methods [48].

When using *Agrobacterium*, the introduced DNA (foreign gene) is incorporated into the bacterial plasmid. Bacteria carrying a chimeric plasmid infect plant cells and transfer the required DNA into them [49].

The second method is bioballistics, which consists of bombarding plant cells with DNA-coated metal particles. In both cases, the DNA that has got into the cell is incorporated into its chromosomes. Then the cell divides, and the whole plant is regenerated from it. The essence of the method for creating transgenic plants is that DNA is deposited on microparticles, which are carriers of a chemically inert metal (gold, tungsten, platinum or ice), 0.6–1.2 μm in size. Then these are compressed with a powder charge helium or an electric field and are accelerated to high speeds (1000 m/s), and a stream of particles is briefly directed to the cell surface. Some of them penetrate into cells and enter the nucleus, where they can be incorporated into the plant chromosomes [50].

At first, embryogenic suspension cell culture was widely used as a target for ballistic transfection to obtain transgenic plants. The low degree of aggregation of the suspension ensured the likelihood of microparticles with DNA entering all target cells. However, the transformation rate was very low; for example, to obtain 1 transgenic plant, 7 to 10 dishes of embryogenic suspension culture of maize were bombarded. The transformation efficiency for different maize cell cultures in experiments ranges from 1 to 3% (after optimization of the method). In some cases, it reaches 18.1%, but not all plants expressed the complete construction of the introduced genes. However, the number of copies of the introduced gene per plant genome during bombardment is rather uncertain, and many of these copies are presented in the form of incomplete sequences, which can lead to undesirable consequences, including the silencing of genes [51].

The disadvantage of the ballistic method is the limitation of the size of the introduced genetic constructs. As a rule, it is possible to transfer the DNA of no more than 10 thousand base pairs. Larger plasmids do not adhere well to metal particles or are destroyed during bombardment. Of all these methods, the *Agrobacterium* method has received the greatest practical application. Other methods have not found wide application for a number of reasons. The use of *Agrobacterium* has advantages over the bioballistic method since it increases the proportion of stable transformation events, can deliver large DNA segments and does not require special ballistic equipment [52].

Agrobacterial plant transformation is the use of *A. tumefaciens*, or, more rarely, *A. rhizogenes*, bacteria as vectors (DNA carriers). It is based on the ability of the pathogenic organism *Agrobacterium* to transfer certain DNA fragments from its Ti plasmid into plant cells. Due to this, native strains of *Agrobacterium* are able to insert into the plant genome various genes responsible for the synthesis of endogenous phytohormones, opines-derivatives of amino acids and sugars. As a result of the expression of transferred genes, tumors or overgrown roots are formed in which various types of opines are synthesized, being a nutrient medium for *Agrobacterium*, but not absorbed by plant cells [53].

Further studies showed that Ti plasmids can act as a biological vector for transferring large DNA fragments (up to 50 kbp) since they have all the components necessary for this [53]:

− a set of virulence genes (VirA, B, C, D, E and G), the coordinated action of which ensures the transfer and integration of the transferred DNA (T-DNA)
− conservative 24-nucleotide direct repeats that flank T-DNA and act as recognition signals of the DNA transfer system
− a T-DNA region that can be replaced with a gene of interest to researchers
− ori-site responsible for replication of the Ti plasmid within *Agrobacterium* [54]

In addition to vir−genes, a group of genes localized in the bacterial chromosome is involved in the operation of the T-DNA transfer apparatus and in ensuring the virulence of *Agrobacterium*. The genes chv A, chv B, psc A and exo C are required for the attachment of *Agrobacterium* to the plant cell wall. As a result of mutations at these loci, avirulent or attachment defective strains of *Agrobacterium* are obtained. However, the oncogenicity and large size of the native plasmid did not allow its use for direct cloning and the transfer of foreign genes [55].

These shortcomings had been overcome in the early 1980s. In 1983, several research groups—one from the University of Washington (USA), the second from the Max Planck Institute for Plant Breeding Research (Germany) and a third from the Ghent University (Belgium)—at once performed the removal of genes that cause tumors using traditional DNA recombination technology. These genes were replaced by genes for kanamycin resistance, as a result of which transformed plant cells acquired antibiotic resistance [56]. Thus, it became possible to obtain a phenotypically normal fertile plant with the expression of foreign genes.

Initially, cointegrative systems of vectors (*cis* vectors) were created in which most of the T-DNA was replaced by a DNA region that had a region of homology with cloning vectors capable of replicating in *E. coli* cells. Thus, in *Agrobacterium* cells, recombination occurs between the homologous region of the modified Ti plasmid (vir helper) and the homologous region of the intermediate vector *E. coli*. A hybrid Ti-plasmid is formed between the native T-DNA boundaries, where there is a complete co-integrative vector of *E. coli* pBR322 containing genes for transfer into plants. The bacterial selectable marker of the *E. coli* plasmid makes it possible to maintain Ti cointegrate in *Agrobacterium* because the *E. coli* vector is not capable of autonomous replication in it. As a result, in *Agrobacterium* strains containing these constructs, during transformation, T-DNA is transferred into the plant genome as a result of the action of the vir region in the *cis* position [57].

The binary vector system (*trans* vectors) consists of two vectors: a disarmed Ti plasmid and a vector that autonomously replicates both in *E. coli* and in *Agrobacterium*. The smaller vector (binary) contains genes between the T-DNA boundaries for transferring to plants. Within its T-DNA, there are multiple cloning sites, allowing the insertion of any DNA sequence of interest. This plasmid is transferred by conjugation into an *Agrobacterium* strain containing a large helper plasmid devoid of oncogenicity, which ensures the transfer of T-DNA from a small plasmid into plant cells [58].

As a result, the transfer of foreign DNA from the cloning vector into the plant cell can be performed by the vir region, which functions in the transposition. The first transgenic plant with the expression of a foreign gene was obtained precisely by the agrobacterium method. Compared to artificial transfer systems, such as the widely used biolistic method, the *Agrobacterium* method has two important advantages [59].

Firstly, *Agrobacterium* transfer into the plant genome only the fragment that is located between two definite DNA sequences, whereas, when artificial systems are used, transformed cells also contain gene fragments or plasmid DNA. Second, the *Agrobacterium* method usually leads to the insertion of only one or at least a limited number of copies of the gene and, at the same time, apparently to certain *loci* of chromosomes, while other methods lead to the random insertion of multiple copies, which, in most cases, are linked to each other [59].

When using the *Agrobacterium* method, the frequency of obtaining stable transformants is higher compared to the biolistic method, and regeneration from protoplasts is not required, compared to direct DNA transfer.

For a long time, the main disadvantage of the agrobacterial system was the lack of a technique for agrobacterial transformation of cereals, but later researchers managed to regenerate the stably transformed callus for wheat and rice. Then transgenic corn and rice plants were obtained by the agrobacterium method. The *Agrobacterium* method is the most widely used one of the three most common plant transformation methods. In 2003, the *Agrobacterium* method was used in 57% of all published papers on transformation. While biolistic transformation and the direct delivery of DNA to protoplasts were used in 25% and 14% of articles, respectively [60].

For the first time, the ability to infect maize with *Agrobacterium* was shown by Grimsley, in which the cDNA of the maize streak virus was delivered to the plant by *A. tumefaciens*, and the plant showed signs of systemic infection. Gould inoculated the stem apices with *Agrobacterium* and produced several transgenic plants. Shen observed β-GUS in maize cells delivered using *Agrobacterium* [60].

The gene for β-glucuronidase (β-GUS) was used as a reporter gene for visualizing the results of transformation, which gives the transformed cells the ability to cleave the substrate X-Gluc (5-bromo-4-chloro-3-indolyl-β-D-glucuronic acid). This process is accompanied by the release of the compound, which, under the influence of atmospheric oxygen, acquires a blue color.

The identification of opine synthase activity in extracts (lysopine dehydrogenase, nopaline dehydrogenase) can serve as evidence that T-DNA has entered the plant cell and is expressed. They are expressed only in the tissues of the infected plant and not in the host bacteria. However, registration by opine tests is difficult and can be complicated by the presence of other substances. Various genes serve as markers for the selection of transformants. The most frequently used genes are nptII, which ensures cell resistance to the antibiotics neomycin and kanamycin, and hpt, which encodes phosphoacetyltransferase, and provides resistance to the herbicide bialaphos (phosphinotricin, PPT), which inhibits glutamatesynthase [61].

Mannose was also used as a selection agent, and phosphomanno isomerase (pmi) was used as a marker gene. This enzyme encodes the conversion of mannose-6-phosphate to fructose-6-phosphate. When mannose is the main or only carbon source, mannose-6-phosphate accumulates in the cells. It inhibits phosphoglucoisomerase, blocking glycolysis, and also deprives cells of orthophosphate, which is necessary for the synthesis of ATP. As a result, mannose, while not toxic in itself, inhibits root growth and respiration and also hinders the formation of embryogenic callus. Cells transgenic by the pmi gene are capable of using mannose as a carbon source.

When transforming, the choice of tissue to be infected is important. Since the goal is usually to produce a whole plant from tissue, cell cultures from which regeneration is possible should be used. In this case, the transformation of protoplasts is ineffective since the regeneration from protoplasts of maize plants is very low. Therefore, embryos, usually zygotic, are used as an object; the transformation of somatic embryos was tested. Callus tissue, which can also be used as an object, develops from the tissues of the flap of immature zygotic embryos on a nutrient medium [15,19,41].

## 5. The Use of Agrobacteria in the In Vitro Production of Callus, Suspension Cells and Root Cultures

In most cases, when producing in vitro callus, suspension cells and root cultures, it is not possible to accumulate and isolate a sufficient number of important metabolites from plant materials. One of the pivotal and encouraging events was the discovery and use of the method of gene transformation of plants using soil bacterium *Agrobacterium rhizogenes* [30].

The agrobacterial transformation of plant roots made it possible to obtain secondary metabolites that are important for medical use: alkaloids, coumarins, phenolic compounds and a number of others [36]. Plant research in this direction is especially relevant.

For more than 20–25 years, scientific research has been actively developing in the world to obtain products of primary and secondary origin in the modified roots of many dicotyledonous and monocotyledonous plants using *Agrobacterium rhizogenes*. At the very beginning of the twentieth century, the phenomenon of hairy roots in apple trees was studied.

*Agrobacterium rhizogenes* is a gram-negative soil bacterium *Rhizobiaceae*, similar to *Agrobacterium tumefaciens*. *Agrobacterium rhizogenes* cells often carry large plasmids called Ri plasmids, which are very similar to Ti plasmids. By the early 1980s, scientists established that a special plasmid is responsible for the virulence of *A. rhizogenes* strains [52,62], which was named Ri by analogy with the Ti plasmid of *A. tumefaciens*, which was named in this way earlier [14].

*Agrobacterium rhizogenes* bacteria carrying Ri plasmids can induce neoplastic root growth in a variety of plants known as hairy roots. After contact with the plant, a part of the plasmid (T-DNA) is transferred into the nuclear genome of the host cells and expressed, causing the constant proliferation of these cells and the accumulation of opines in them.

One of the genetic features of Ri plasmids is the presence of several rol genes (from the English “root locus”): rolA, rolB, rolC and rolD. Recently, a group of Russian authors published a review in which each of these genes was considered in detail, and suggestions were made regarding the role of each of them in the formation of hairy roots [22]. Earlier, other scientists examined the rol-genes of *A. rhizogenes* for their effect on the synthesis of secondary metabolites in plants [38]. They suggested that some of the rol genes can suppress the immune response, thereby allowing this phytopathogenic bacterium to infect an infected plant [52,62].

Currently, scientists around the world use at least 90 strains of *A. rhizogenes* on 400 plant species in fundamental and applied research to obtain hairy roots, but the use of two agropine strains, ATCC15834 and A4, is most described in the scientific literature. They are distinguished by a high degree of virulence. Long-term efforts of breeders have turned plants into food and medicinal crops, which are the main sources of biologically active substances and possess antioxidant, antiviral, antimutagenic and other properties [53,63].

The methods of traditional breeding of medicinal crops cannot meet the growing needs of mankind in this direction. Recent advances in plant biotechnology open up new opportunities for improving yields and quality and for making the production of medicinal plants cheaper [53,63]. One of the methods for stimulating the synthesis of biologically active components is the transformation of a plant with the bacterium *Agrobacterium rhizogenes* to obtain a culture of genetically transformed roots (hairy roots), which can be a stable system for the production of biomass with a high level of synthesis of secondary metabolites and for obtaining pRi T-DNA regenerant plants producing secondary compounds in larger amounts than intact plants [64].

As it turned out, in this case, two groups of genes are transferred and inserted into the plant genome: the products of the first group interfere with the normal metabolism of the plant and contribute to the growth of the tumor, while the products of the second group synthesize opines, substances not needed by the plant but used as food by bacteria.

Scientists have modified agrobacteria in such a way that they transfer the required genes into plants instead of their own [65].

A certain range of agrobacteria is used more often in research works on the formation of hairy roots for a number of reasons, among which, one of the main reasons is the high virulence of these strains. Another reason for the use of specific strains in the experiments of different authors is their presence in the collections of local institutes.

All *A. rhizogenes* strains can be subdivided into four groups according to the type of Ri plasmids present in them, encoding the corresponding opines: agropine, mannopine, cucumopin and mykimopin. Table 1 lists a number of commonly used strains for which the types of opines synthesized are known.

A review article has been published in which many issues of opine metabolism in both *A. tumefaciens* and *A. rhizogenes* are considered in rather detailed comparative terms [66]. The virulence of *A. rhizogenes* strains is usually assessed using carrot root disks, which give a fairly quick and accurate response in the form of the formation of hairy roots in the pericycle zone with different intensities when such disks 5 mm thick are placed on nutrient agar. Moreover, a number of strains are active depending on the side on which the carrot root disc is placed on the agar (apical or basal). Although such a test provides only primary information about the infection and the growth rate of hairy roots, it also allows *A. rhizogenes* strains to be divided into so-called polar and non-polar strains. Agropine strains are non-polar and are generally considered to be more virulent and capable of infecting more species, but there are plant species that are more susceptible to attacks, such as cucumopine or some other types of *Agrobacterium*.

Not all authors provide information on the types of opines synthesized by *A. rhizogenes* strains in their articles. At the same time, in a number of cases, references are made to their own previous papers or to articles by other authors, which, logically, should have contained more information about the strains but, in fact, usually do not. In many articles, the authors, indicating the origin of the strains, thank their colleagues for sending them the strains, but they often send not the strains they found themselves but those that were right at their fingertips. All this creates a rather serious confusion, especially when trying to comparatively evaluate the effectiveness of the infection of plants with certain strains used in some articles.

Altamura’s work made a great contribution to the systematization of the data on the strains of soil bacteria [67]. The authors analyzed the “popularity” and prevalence of *A. rhizogenes* strains for the production of hairy roots in the world.

**Figure 1:**
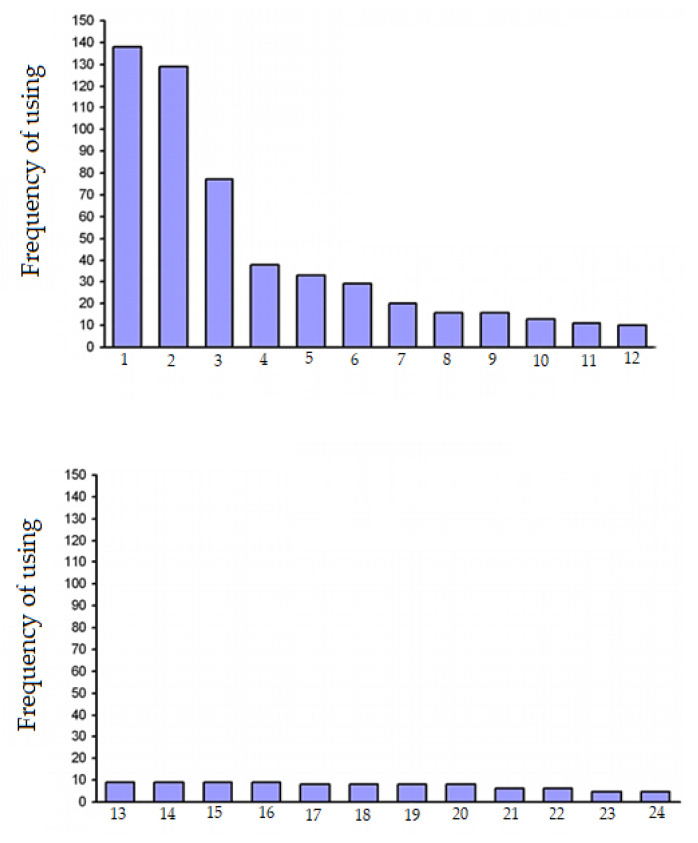
Frequency of use of *A. rhizogenes* strains in the world: 1—ATCC15834; 2—A4+ATCC43057; 3—LBA9402; 4—R1000; 5—K599+NCPPB2659; 6—NCIB8196; 7—R1601; 8—C58C1; 9—NCPPB1855; 10—MAFF301724; 11—A4T; 12—TR7; 13—A4RS; 14—ARqual; 15—TR101; 16—ATCC11325; 17—1334; 18—TR105; 19—MSU440; 20—MTCC532; 21—HRI; 22—LMG150; 23—A13; 24—R1600 (this is a modified figure from ref. [68]).

Figure 1 shows the frequency of use of *A. rhizogenes* strains in the world, calculated from 424 analyzed research articles published by different authors over forty years. Since, in a significant amount of research, the authors used more than one strain, the total number of strains significantly exceeds the number of analyzed articles. Figure 1 shows that, in the overwhelming majority of cases, the strain ATCC15834 was used (another rarer naming is A4, which also used in some articles, and it is sometimes referenced as ATCC43057). Thus, the ATCC15834 strain was used in almost every third work. The A4 strain is mentioned a little less often. Approximately one in six articles reports the use of the LBA 9402 strain. Three more strains (R1000, K599 and NCIB8196) were used slightly more often than the others. In addition to the strains mentioned in Figure 1, we present the names of other *A. rhizogenes* strains used for the formation of hairy roots.

The growth productivity of in vitro callus, suspension cells and root cultures, depending on the strain of *Agrobacterium* and the temperature of cultivation, is presented in Table 2 [69].

Thus, Christey and Braun’s studies of *Agrobacterium* AR10, DC-AR2 and TR104/ATCC13333 were used. The articles also mention strains AR12, A4RSII, R1200 and TR102/ATCC13332 (three per strain). Strains A5, A7, TR107, LBA920, LBA9435, AR25, A4PC, LMG63, AM8703, A2/83, A20/83, AR1193, NIAES1724 and MTCC2364, were used in two research studies each. A very large number of analyzed strains were used by the authors only once [69].

The authors managed to count 90 strains used to obtain hairy roots. At the same time, the references to the *A. rhizogenes* strains used in articles can be both the original names of the strains and the inventory numbers assigned to them by different collections, which makes it difficult to compare the agrobacteria used by different authors. In addition, the numbers are not always accompanied by letter indices, which could create a better understanding of which strains were used. However, the same letter indices do not always indicate the relationship and common origin of the strains. Thus, for example, the A4 strain was isolated by P. Ark in the USA, and the A5 and A13 strains were isolated in Japan by Professor M. Mii.

A number of articles study the degree of virulence of different strains upon the induction of the growth of hairy roots in various plants [46,56,61].

Three strains (15834, A4 and K599) were tested for their effectiveness in the formation of hairy roots in 14 grape species [70]. The results indicate that the first two strains were equally effective, while the K599 strain showed the worst results. When generating hairy roots in one of the species of madder *Rubia akane*, Korean authors used several *A. rhizogenes strains*, including 13333, 15834, R1000, R1200 and R1601, of which the latter showed the best results in terms of virulence and productivity of the growth of such roots [71]. Two strains of *A. rhizogenes*, 15834 and LBA 9402, were used in the induction of hairy roots in the opium poppy, which is considered difficult to transform [67]. After five weeks, strain LBA 9402 produced hairy roots in 80% of cases. Five different strains of *A. rhizogenes* (31978, AR12, 43057, A4 2 and A13) were tested to obtain hairy roots in *Solanum mammosum* [72]. Strains AR12 and A13 showed a very rapid formation of hairy roots in this plant species in just six days, while strain 31978 provided the most noticeable increase in root biomass.

When producing a callus cell culture of *Artemisia annua* with a number of *A. rhizogenes* strains (A4, 15834, K599, LBA9402, 9365 and 9340), the highest yield of such a secondary metabolite as artemisinin was observed in the culture of hairy roots obtained with the strain LBA 9365 [73]. In one of the works, the production of hairy roots in two species of henbane was carried out using strains A4 and LBA 9402 [74]. The second strain provided more than a threefold increase in the production of atropine compared to the A4 strain. The above information indicates that, in some plants, some strains exhibit the lowest efficiency, while they can be extremely aggressive in other species. Thus, for the successful formation of root cultures of any plant species, it is desirable for an experimenter to have many strains of *A. rhizogenes*, moreover differing in opine types, at their disposal.

Ooi et al. obtained a stably growing culture of genetically transformed hairy roots and regenerant plants of *Digitalis purpurea* L. using the *Agrobacterium rhizogenes* 15834 strain [74]. It was found that, as a result of spontaneous regeneration from transformed roots on a medium with a low nitrogen content under conditions of incubation in the light, Ri transformants of *Digitalis purpurea* L. were obtained. The root regenerants were characterized by enhanced rhizogenesis and effective weight gain. PCR analysis confirmed the rolB gene transfer from *A. rhizogenes* T-DNA into the plant genome, and HPLC analysis showed that the roots synthesize the entire complex of secondary compounds typical for the intact plant in an amount greater than in callus culture but insufficient for industrial cultivation. Regenerated plants synthesized the entire spectrum of secondary compounds, and their content is 58% higher than in intact plants. This confirms the data that the introduction of the transforming region of *A. rhizogenes* 15834 into the nuclear genome of plants not only changes the morphology of plants but also causes a change in their secondary metabolism.

The authors of [75] carried out work aimed at *A. rhizogenes*-mediated transformation of periwinkles (*Vinca minor*), obtaining a hairy root culture, plant regeneration and primary analysis of the resulting lines for the content of biologically active substances. *A. rhizogenes* strains A4, R-1601, 8196 and 15834, transformed with the binary vector pBin35S-GFP, containing marker genes gfp, a green fluorescence protein reporter gene, and npt II, were used.

The research resulted in revealing the frequency of transformation for each strain (Table 3).

As a result of the studies, it was found that *A. rhizogenes* R-1601 is the most effective agrobacterial strain.

## 6. Conclusions

Modern methods of obtaining and using callus, suspension cells and root cultures of medicinal plants in vitro are studied. The history of the in vitro cultivation of callus, suspension cells and root cultures is described. The advantages of using the hairy root culture as a producer of biologically active substances are presented. Genetic engineering and biotechnology in the in vitro production of callus, suspension cells and root cultures proved to be promising methods for global studies of the problem of accumulation of biologically active substances in tissues and organs of intact plants. It is established that the use of *Agrobacterium* in the in vitro production of callus, suspension cells and root cultures is the most promising modern method of cultivating plant tissues and organs.

## Figures and Tables

**Table 1:** *Agrobacterium rhizogenes* strains producing different opines.

Agropine	Mannopine	Cucumopine	Mikimopine
ATCC15834	LMG63	K599 *	MAFF30
A4	LMG150	NCPPB2588	1724
LBA9402	NCIB8196	NCPPB2659	MAFF02-10266
HRI	TR7	NCPPB2657	A13 **
NCPPB1855	TR101		A5
R1601	TR105		A6

* K599 and NCPPB2659 are the same strain, and the original name K599 is used in articles more often. However there is a considerable number of articles using the NCPPB2659 naming as well. ** The same should be said about *Agrobacterium* A13 and MAFF02-10266, focusing only on the fact that, as a rule, Japanese authors are using its accession number in the collection of the Japanese Ministry of Agriculture, Forestry and Fisheries.

**Table 2:** Dependence of the growth productivity of callus, suspension cells and root cultures in vitro on the strain of *Agrobacterium* and the temperature of cultivation.

*A. rhizogenes* Strains	Cultivation Temperature, °C	In Vitro Tissue Growth Productivity, %
ATCC15834	21	80
A4+ATCC43057	26	73
LBA9402	20	78
HRI	18	21
NCPPB1855	30	70
R1601	30	58
R1000	33	72
LMG150	34	40
NCIB8196	32	79
TR7	27	48
TR101	23	49
TR105	25	51
K599+NCPPB2659	30	73
C58C1	27	58
A4T	31	41
MAFF301724	18	59
A4RS	18	39
A13	21	66
ARqua1	22	42
ATCC11325	28	46
1334	23	47
MSU440	31	52
MTCC532	33	50
R1600	28	61

**Table 3:** Transformation frequency (%) by different strains of *A. rhizogenes.*

Strain	*V. minor*		*C. roseus*
	YEB Medium	YEB Medium + *N. tabacum* Extract	
A4	2.3 ± 0.9	9.0 ± 0.3	30.4 ± 0.8
R-1601	10.0 ± 0.7	35.6 ± 0.5	54.3 ± 0.5
8196	0.0 ± 0.0	5.8 ± 0.7	24.2 ± 0.7
15834	3.4 ± 0.5	14.3 ± 0.9	20.7 ± 0.9
