# Correction: Babich et al. Modern Trends in the In Vitro Production and Use of Callus, Suspension Cells and Root Cultures of Medicinal Plants. *Molecules* 2020, *25*, 5805

**DOI:** 10.3390/molecules27175513

**Published:** 2022-08-27

**Authors:** Olga Babich, Stanislav Sukhikh, Artem Pungin, Svetlana Ivanova, Lyudmila Asyakina, Alexander Prosekov

**Affiliations:** 1 Institute of Living Systems, Immanuel Kant Baltic Federal University, A. Nevskogo Street 14, 236016 Kaliningrad, Russia; 2 Department of Bionanotechnology, Kemerovo State University, Krasnaya Street 6, 650043 Kemerovo, Russia; 3 Natural Nutraceutical Biotesting Laboratory, Kemerovo State University, Krasnaya Street 6, 650043 Kemerovo, Russia; 4 Department of General Mathematics and Informatics, Kemerovo State University, Krasnaya Street 6, 650043 Kemerovo, Russia; 5 Laboratory of Biocatalysis, Kemerovo State University, Krasnaya Street 6, 650043 Kemerovo, Russia

In the original publication [1], reference [68] was not cited. The citation has now been inserted in Figure 1 caption, The correct Figure 1 caption should read: 

**Figure 1.** Frequency of use of *A. rhizogenes* strains in the world: 1—ATCC15834; 2—A4+ATCC43057; 3—LBA9402; 4—R1000; 5—K599+NCPPB2659; 6—NCIB8196; 7—R1601; 8—C58C1; 9—NCPPB1855; 10—MAFF301724; 11—A4T; 12—TR7; 13—A4RS; 14—ARqual; 15—TR101; 16—ATCC11325; 17—1334; 18—TR105; 19—MSU440; 20—MTCC532; 21—HRI; 22—LMG150; 23—A13; 24—R1600 (this is a modified figure from ref. [68]).

Newly added reference [68] should read: Kuluev, B.R.; Vershinina, Z.R.; Knyazev, A.V.; Chemeris, D.A.; Baymiev, A.K.; Chumakov, M.I.; Baymiev, A.K.; Chemeris, A.V. Plant hairy roots are important instrumentation for researchers and powerful phytochembiofactory for manufacturers. *Biomics* **2015**, *7*, 70–120.

With this correction, newly added reference [68] and the order of some references has been adjusted accordingly.

The authors would like to apologize for any inconvenience caused to the readers by these changes. The original article has been updated.
